# Side-by-Side Comparison of Compensation Beads Used in Polychromatic Flow Cytometry

**DOI:** 10.4049/immunohorizons.2300066

**Published:** 2023-12-06

**Authors:** Debajit Bhowmick, Sara K. Lowe, Michelle L. Ratliff

**Affiliations:** * Flow Cytometry Facility, Brody School of Medicine, East Carolina University, Greenville, NC; † Department of Microbiology and Immunology, Brody School of Medicine, East Carolina University, Greenville, NC

## Abstract

Compensation or unmixing is essential in analyzing multiparameter flow cytometry data. Errors in data correction, either by compensation or unmixing, can completely change the outcome or mislead the researchers. Owing to limited cell numbers, researchers often use synthetic beads to generate the required single stains for the necessary calculation. In this study, the capacity of synthetic beads to influence data correction is evaluated. Corrected data for human peripheral blood cells were generated using cell-based compensation from the same cells or bead-based compensation to identify differences between the methods. These data suggest that correction with beads on full-spectrum and conventional cytometers does not always follow the basic flow compensation/unmixing expectations and alters the data. Overall, the best approach for bead-based correction for an experiment is to evaluate which beads and fluorochromes are most accurately compensated/unmixed.

## Introduction

To successfully perform a polychromatic flow cytometry experiment, nothing is more important than correct compensation. Previously it was demonstrated that when there were no spectral differences between experimental samples and compensation controls, beads could be used to compensate cells (1–3). Maciorowski et al. (4) advised users to check single-color stained cells and single-color stained beads to identify which produces better compensation and use that for the rest of the experiment. However, this guidance is mostly qualitative, not quantitative, and not based on any comprehensive side-by-side comparison of beads and cells. This is further complicated by the large number of compensation bead options currently on the market.

Spillover in both full-spectrum and conventional platforms from the fluorochromes needs to be rectified using either unmixing (where the number of detectors is higher than the number of fluorochromes) or compensation (where the number of detectors is the same as the number of fluorochromes), respectively.

Unmixing/compensation (hereafter referred to as correction/corrected/correct) is often an issue with these experiments due to the disparity of effectiveness between beads and cells. In most cases, users have not performed all of the controls to address this issue. Furthermore, the published literature does not demonstrate side-by-side cell-based versus bead-based corrected data.

It is known that bead-based compensation sometimes does generate unexpected/wrong outcomes. Interestingly, recent studies involving high parameter panels used cells, not beads, to generate single stains (5–7). These studies used full-spectrum Aurora. It is becoming more evident that full-spectrum machines are more sensitive than photomultiplier tube–based conventional machines. It appears that cell-based single stains are gaining attraction for these full-spectrum machines. Of course, the use of cells is limited to the availability of sufficient numbers of cells to for single-color controls and experimental staining. Researchers have compared beads and cells for compensation in the past, leading to direct publication of the comparisons or using mixed cells/beads for compensation matrices (Refs. 8–12 and (Y. Shevchenko, I. Lurje, F. Tacke, and L. Hammerich, manuscript posted on bioRxiv, DOI: 10.1101/2023.06.14.544540), but all of these works are limited to one or two types of bead versus cells. Interestingly, many university flow cytometry core facilities and compensation bead vendor pages make mention that the compensation generated using beads is not always perfect. A CYTO (International Society for Advancement of Cytometry) poster presented in 2019 (13) and a poster from Friend et al. (14) at the 2023 American Chemical Society conference studied the same concept in a limited manner. Taken together, these findings suggest that the concept that there are differences between compensation beads and cells is a common issue, but the differences between the many different beads currently on the market have not been investigated, suggesting a general assumption that the differences between the bead sets are negligible. To investigate the merits of this likely assumption, we compared eight of the available beads on the market at the time of initiation with human peripheral blood cells, with all samples run in the same day, and data were compared systematically. To minimize additional caveats to the comparisons, the methods for comparison and panel design were not overly complicated.

These data suggest that experimental design should include additional time to address correction controls and what should be the appropriate controls for each experiment. At the same time with this work, we want to bring the attention of the wider audience to the topic that we need to spend resources and time to fix this issue. Highly experienced flow experts from both academia and industry need to investigate this problem closely and work together to solve it. If left unanswered, this phenomenon will ultimately cause massive data reproducibility problems, even with the honest efforts from the user’s side.

## Materials and Methods

### Cells

PBMCs from two healthy male adults were isolated from a leukocyte reduction system chamber purchased from the Oklahoma Blood Institute with Lymphoprep (STEMCELL Technologies). Isolated PBMCs were resuspended in serum-free cell freezing media (Bambanker, Bulldog Bio) and stored at −80°C for later use. Oklahoma Blood Institute obtained relevant informed consents for blood product use for research purposes. Because the chambers are a byproduct of platelet donation and do not cause additional risk to the donor, the East Carolina University Institutional Review Board does not require a board review of protocols. Protocols were approved by East Carolina University Institutional Biosafety Committee (protocol 01-19). All methods were carried out in accordance with relevant guidelines and regulations.

### Ab capture bead or compensation bead

Eight different beads (details in Table I) from Thermo Fisher Scientific (UltraComp, OneComp, AbC Total), BD Biosciences, Beckman Coulter (VersaComp), Miltenyi Biotec (MACS), Spherotech (COMPtrol), and Slingshot were used. Only the positive capture beads from BD Biosciences, Beckman Coulter, MACS, COMPtrol, and AbC Total were used for this work. Unlabeled positive capture beads (without Abs) were used as the negative control to ensure the same autofluorescence (AF) between Ab-bound and unbound beads. Slingshot, OneComp, and UltraComp provide only one vial with blank and positive beads. These three unstained beads were run as the negative control.

### Ab–fluorochrome conjugate

All experiments to establish a median mismatch index (MMI) were done using a human CD4 evaluation kit (BD Biosciences, 566352) except for the Nova Yellow 690, which was purchased from Thermo Fisher Scientific (H001T03Y05). The following fluorochromes were used: BUV615, BUV661, and BUV737 (under 355-nm excitation); BV421, BV650, and BV711 (under 405-nm excitation); FITC and BB700 (under 488-nm excitation); PE-CF594, PE-Cy5, and Nova Yellow 690 (under 561-nm excitation); and allophycocyanin and Alexa Fluor 700 (under 640-nm excitation). DAPI, CD14 PE-Cy5 (BioLegend, 301863), CD3 allophycocyanin-R700 (BD Biosciences, 565120), CD4-allophycocyanin (BD Biosciences, 566915), CD8-BV786 (BioLegend, 344740), CD19 BB700 (BD Biosciences, 566397), and CD45RA-BV711 (BioLegend, 304137) were used to generate a small panel for testing the effect of compensation beads on biological interpretation in terms of the position of these populations on the plot.

### Staining of PBMCs and beads

The following steps detail the staining procedures used. 1) Cryopreserved PBMCs were thawed in a 37°C water bath, shaking continuously until the suspension was almost thawed. When only a small amount of ice was left in the cryotube, the suspension was transferred to a 15-ml conical tube and 10 ml of 37°C media containing 2 U/ml endonuclease was added dropwise to the suspension. Following centrifugation at 400 × *g* for 10 min at room temperature, cells were washed with 10 ml of 37°C media, filtered gently through a 40-μm cell filter, and counted. 2) Cells (1 × 10^6^) were dispersed into flow cytometry–appropriate tubes, pelleted again at 400 × *g* for 5 min at 4°C, and resuspended in 100 μl of staining buffer (BD Biosciences, 554656). In the case of beads, seven drops of beads were added in a FACS tube, followed by the addition of stain buffer to reach the volume of 1400 µl. Then, the beads were equally distributed in 14 tubes. 3) One hundred fifty and 500 ng of CD4 Abs were added to cells and beads, respectively (at saturation). 4) The samples were stained at 4°C for 30 min in the dark. 5) Cells/beads were washed twice in 3 ml of cold stain buffer, then 5 min of centrifugation at 400 × g (acceleration 9, deceleration 7). 6) Both cells and beads were resuspended in 200 μl of staining buffer and analyzed immediately on both cytometers.

### Flow cytometers

Five-laser Cytek Aurora and four-laser BD FACSAria Fusion cell sorters were used for data generation. Details of the Aurora configuration can be found in Brandi et al. (5). Configuration details of the sorter are available on our Web site (https://medicine.ecu.edu/flow-core/instruments-configuration/). The Aurora was configured to gain values recommended by Cytek assay settings. The Fusion was configured using photomultiplier tube voltages recommended by cytometer setup and tracking beads instructions provided by the manufacturer. Quality control assays recommended by the manufacturer were run before every experiment.

### Software

SpectroFlo 2 and FACSDiva 8 were used for data acquisition. Conventional flow data were analyzed using FlowJo 8, and full-spectrum data were analyzed using SpectroFlo 2. Graphs were prepared using either Microsoft Excel or GraphPad Prism. FlowJo 8 was used to generate the t-distributed stochastic neighbor embedding (tSNE) plots. After each unmixing application, full stain cell data were exported, cleaned, downsampled to the same number, concatenated, and then opt-SNE was run.

### Data collection

Ten thousand events as the main population in the scatter plot were collected for compensation bead analyses. At least 50,000 lymphocytes in the scatter plot were collected for cell analyses. A low flow rate was used for Aurora data collection, but a varying flow rate was used for Fusion collection. Data are available on FlowRepository (accession no. FR-FCM-Z6KC). Except for the seven-color panel for tSNE work, all data were repeated in Aurora and Fusion. We process data from both machines. The output from both machines is similar. All of the plots in this article were created using Aurora data only. Seven-color panel data were only acquired in Aurora. We ensured that the data in both machines were within the linearity range of each detector. The baseline cytometer setup and tracking beads report provides the maximum and minimum linearity of each detector and can confirm that our data are within this range. In the case of Aurora, manufacture recommended quality control does not provide this range; however, service engineers do check this range during preventive maintenance. We can confirm that our Aurora data are within the linearity range.

### Dataset used

External data from optimized multicolor immunofluorescence panel articles were used for additional analyses (15–18).

### Compensation/unmixing strategy

We choose to use single-stained samples to evaluate accuracy of compensation or unmixing as shown by Verwer (19) in his 2002 white paper. The “negative” cells or beads in the single-stain tubes were not used for calculation because they always have some nonspecifically bound Ab, which changes the fluorescence intensity level of the reference control, leading to erroneous calculations. This will violate the rule of same AF. Separate negative controls were used for compensation/unmixing calculations. Compensation matrices and unmixing were autocalculated in the software using bead or cell data and then applied to the cell or bead data. The median fluorescence intensities (MFIs) of the single-stained positive cells were measured and compared with the MFIs of the single-stained positive beads. When the beads had a lower MFI than the cells, the data were excluded for those fluorochromes from the compensation or unmixing calculations. SpectroFlo does allow AF removal, but we have not used this feature in this work.

### Quantifying compensation/unmixing mismatch

**FIGURE 1.**
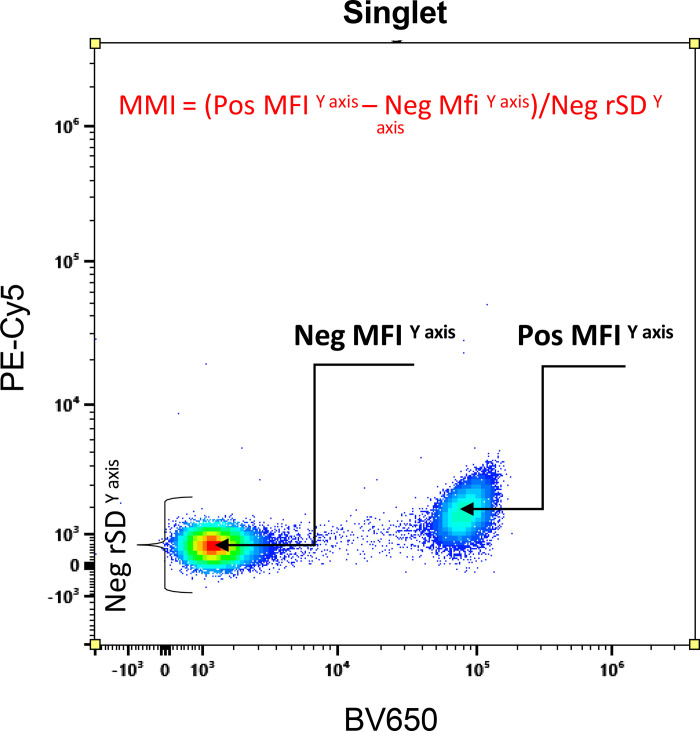
Schematic of MMI calculations. Details of the concept of MMI are shown.

**FIGURE 2.**
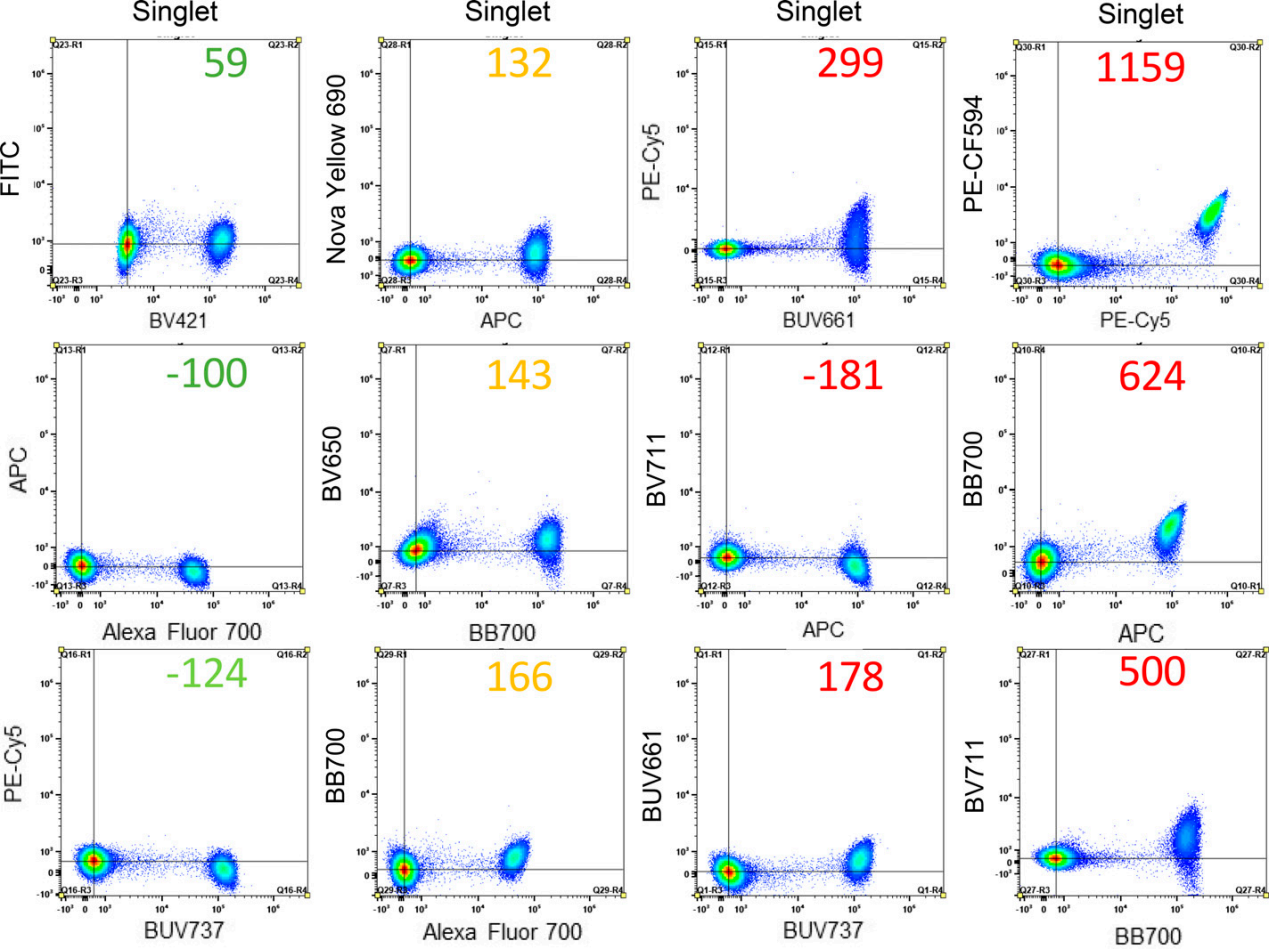
Fluorochrome spillover affects data visualization. PBMCs were stained using anti-CD4 Abs labeled with different fluorochromes, and data were acquired by flow cytometry. Data from selected fluorochromes are shown to demonstrate spillover between channels. Numbers in each plot indicate the MMI for each. Values between −125 and 125 are indicated in green (acceptable), values between −170 and −125 and 125 and 170 are in orange (partly acceptable), and values beyond −170 and 170 are in red (not acceptable). All data shown are from the Cytek Aurora (*n* = 3).

An MMI (see Fig. 1) was used to quantify median mismatch. These are expressed as follows: MMI% = 100[(positive MFI*^y^*^-axis^ − negative MFI*^y^*^-axis^)/negative RSD*^y^*^-axis^], where RSD is the robust SD. The MMI is similar to the secondary stain index (20), but is modified and two times more robust. We made a classification of acceptable and unacceptable median mismatches. These values are classified based on deviation from the median of the negative population on the *y*-axis. Values between −125 and 125 are indicated in green (acceptable), which is a 25% deviation above and below the median; values between −175 to −125 and 125 and 175 are in orange (partly acceptable), which is a 25–75% deviation above and below the median; and values beyond −175 and 175 are in red (not acceptable), which is a deviation >75% above and below the median. Some examples can be found in Fig. 2. At this time there is no quantitative method to describe the median mismatch. The rationale behind these categories or divisions is simple. A median mismatch within 25% of the RSD of the unstained cells (green) was considered acceptable based on their visual appearance, which shows no indication of a double-positive population in a single-stained sample. This insignificant amount of variation does not influence the outcome in a wrong way. In the orange category this deviation is within 25–75% of the unstained RSD. This amount of variation may slightly influence the outcome (as it appeared to be in slightly double-positive populations), but an expert can identify these easily and will not consider the population to be double-positive. Contrary to the red category where the median mismatch lies outside ±75% of the unstained cells, the RSD quite easily appears as a false double-positive. This amount of deviation can easily change the conclusion of the study. Hence, the red category needs to be avoided.

### Generating the fluorescence emission profile

Cytek recommended steps were used to calculate all the spectra using the raw data.

### Ab binding sites on beads compared with cells

Next, the approximate number of Ab binding sites on the beads compared with the binding sites present on cells was calculated, as this could potentially explain why some beads failed the bright and brighter rule. The ΔFMI^cell^ = (MFI positive^cell^ – MFI blank^Cell^) and ΔFMI^bead^ = (MFI positive^bead^ – MFI blank^bead^) were calculated. The number of Ab binding sites on the bead/Ab binding site on the cell is ΔFMI^bead^/ΔFMI^cell^. Table II shows that the number of binding sites can vary within one type of bead. It could be due to the size of the fluorochrome and/or the geometry of the bead surface that allows the Ab to bind. As the clone and Ab amount remain the same, the only variables are the fluorochromes. If the Ab binding to the bead is fluorochrome-independent, then one can expect the same number of binding sites on a specific bead type, which is not the case.

### Effect of compensation beads on biological data interpretation

**FIGURE 3.**
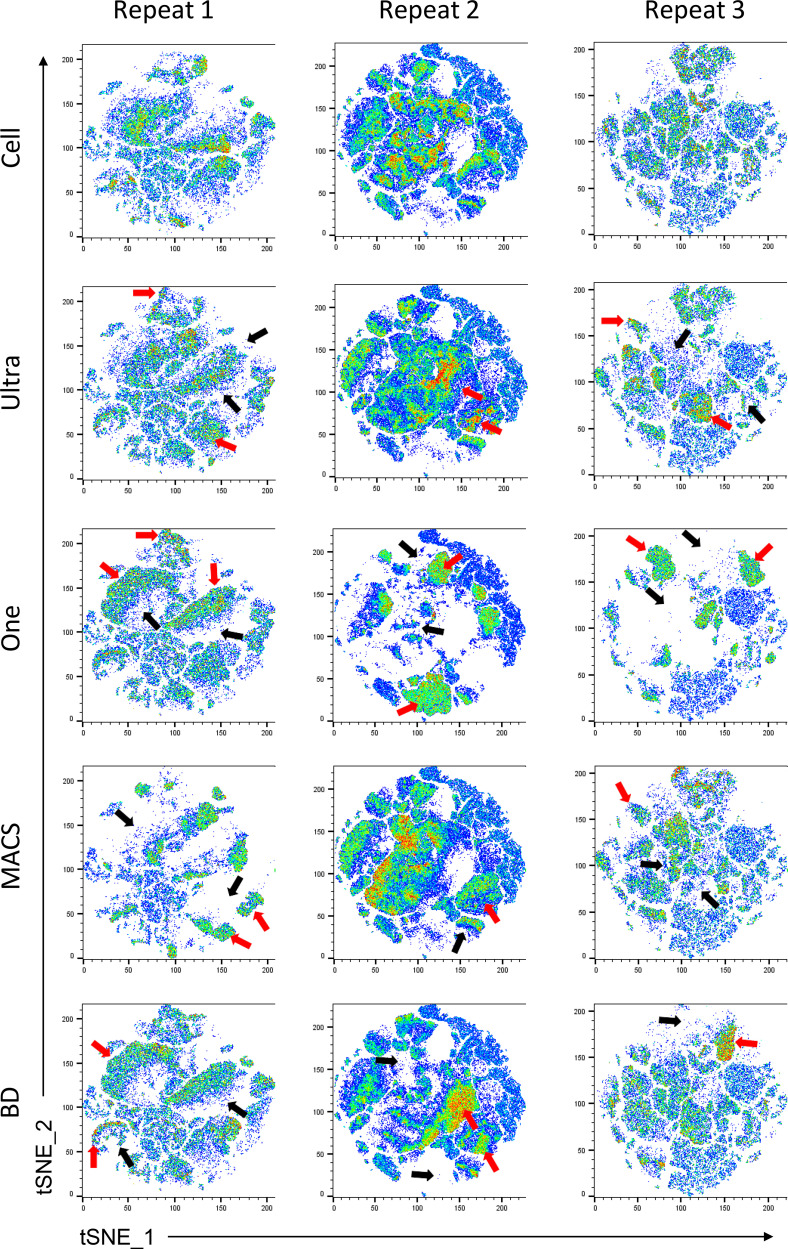
tSNE analyses reveal population differences based on the correction method. PBMC data from Supplemental Fig. 1 were analyzed using tSNE-directed analysis. After applying correction (cells or beads), FCS files were extracted, cleaned, and downsampled to the same number of cells, concatenated, and then optSNE was run using FlowJo 8 and finally segregated into five separate plots (one column). The red and black arrows point to the new populations that appeared due to bead-based correction and the disappearance of the populations present on the cell-based unmixing. Each column represents one repeat.

A small panel using DAPI (2 ng/ml), CD3 allophycocyanin-R700, CD19 BB700, CD4 allophycocyanin, CD8 BV786, CD14 PE-Cy5, and CD45RA BV711 Abs was designed. To unmix the fully stained cell sample, single stains from four different beads (UltraComp, OneComp, MACS, and BD Biosciences) and cells were made: 0.75 µl of each Ab was used for the full stain sample, and 2 µl of each Ab was used for all single-stain preparations. Data were analyzed after applying different unmixing matrices on the fully stained cell sample (Supplemental Fig. 1). Fig. 3 shows the side-by-side comparison of tSNE plots of three repeats using these four beads. One dedicated unstained cell sample was used to unmix the DAPI signal in both cell-based and bead-based unmixing strategies. SpectroFlo software does allow multiple unstained samples as a reference, and we took full advantage of this feature. FACSDiva allows only one universal unstained sample as a reference.

## Results

### Identification of baseline variation of median mismatch

**FIGURE 4.**
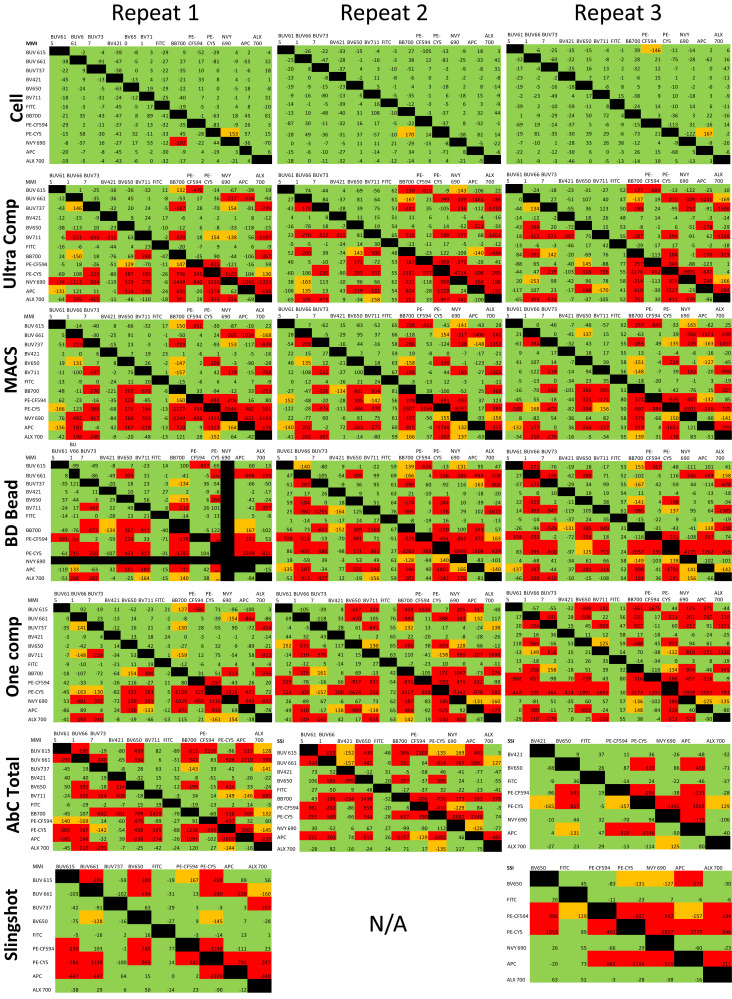
Compensation beads fail to properly correct cell data. PBMCs as in Fig. 2 were corrected by the same cells or compensation beads, also stained as in Fig. 1. The beads that passed bright and brighter were included. Unmixing the cell data using the same cell data are shown in the firs row. Rows 2–7 include the same cell data (columnwise) but were unmixed using different beads as represented on the left. Values between −125 and 125 are indicated in green (acceptable), values between −170 and −125 and 125 and 170 are in orange (partly acceptable), and values beyond −170 and 170 are in red (not acceptable). All data were acquired on the Cytek Aurora and the BD FACSAria Fusion (*n* = 3).

Single-cell samples were compensated/unmixed using the same cell samples (Tables I, II). The spillover matrix is assumed to be perfect, as the same data were used for unmixing calculations and as test samples. For each combination, MMI values were calculated (Fig. 1). Examples of the data plots with MMI values denoting acceptable (green), partially acceptable (orange), and not acceptable (red) spillover are shown in Fig. 2. Gates were placed at the center of the negative population, and MMI values were chosen based on plot visualizations and MMI numbers. These experiments were repeated three times for a full panel (Fig. 3) and single color stains (Fig. 4) with similar results.

**Table I.** Details of the Ab capture bead

	Thermo Fisher Scientific			Miltenyi Biotec: MACS Comp Beads Kit, Anti-Mouse Igκ	BD Biosciences: Anti-Mouse Ig, κ/Negative Control Compensation Particles Set	Beckman Coulter: VersaComp Ab Capture Kit	Slingshot Biosciences: SpectraComp Compensation Beads	Spherotech: COMPtrol Kit, Goat Anti-Mouse Ig (H&L)–Coated Particles
	UltraComp eBeads Compensation Beads	OneComp eBeads Compensation Beads	AbC Total Ab Compensation Beads					
Catalog no.	01-2222-42	01-1111-42	A10513	130-097-900	552843	B22804	SSB-05-A	CMigp-30-5H
Lot no.	2285467 and 2306368	2324808	2502902 and 2372350	52201		1271047	211003	AN05
Vials in kit	1	1	2	2	2	2	1	2

Details of all eight Ab capture beads are presented.

**Table II.**
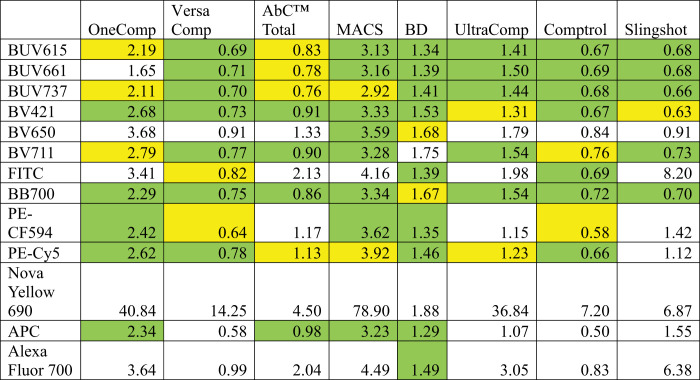
Ab binding sites on beads

The number of Ab binding sites on eight different beads is presented in terms of the number of Ab binding sites on CD4^+^ cells. Green and yellow boxes indicate the values are well within the ±10% and ±10–20% range, respectively. The values of Nova Yellow 690 were not included in the calculation, as they have very poor cell binding. The data are the averages for all three runs.

### Identification of median mismatch when using beads for correction

Next, unmixing was calculated using beads and then applied to the cell data. The first rule for compensation is that the single stains used should be equally bright or brighter than the experimental sample (21). COMPtrol and VersaComp beads consistently failed to show brighter signals than the cells (even though they were stained to saturation). As they failed to qualify with this rule, they were not used to correct cells. AbC Total and Slingshot beads appear brighter for some fluorochromes (such as FITC, PE-CF594, PE-Cy5, allophycocyanin, Alexa Fluor 700), but not all fluorochromes. These fluorochromes were only used to correct cells. BD Biosciences, OneComp, UltraComp, and MACS beads were brighter than cells for all fluorochromes in all three repeats. All fluorochromes of these four bead types were used to correct cells.

Similar to the above cell-on-cell data, MMIs for all combinations were calculated. As shown in Fig. 4 (rows 2–7), many unacceptable median mismatches (red) were observed. A high quantity of red was observed for all beads assessed. The patterns in the matrix were reproducible in all three repeats. Although the patterns are indeed reproducible, there were not perfect overlaps in the individual fluorophores between repeats, indicating some variability in the spectra between independent runs.

### Comparison of spectra

**FIGURE 5.**
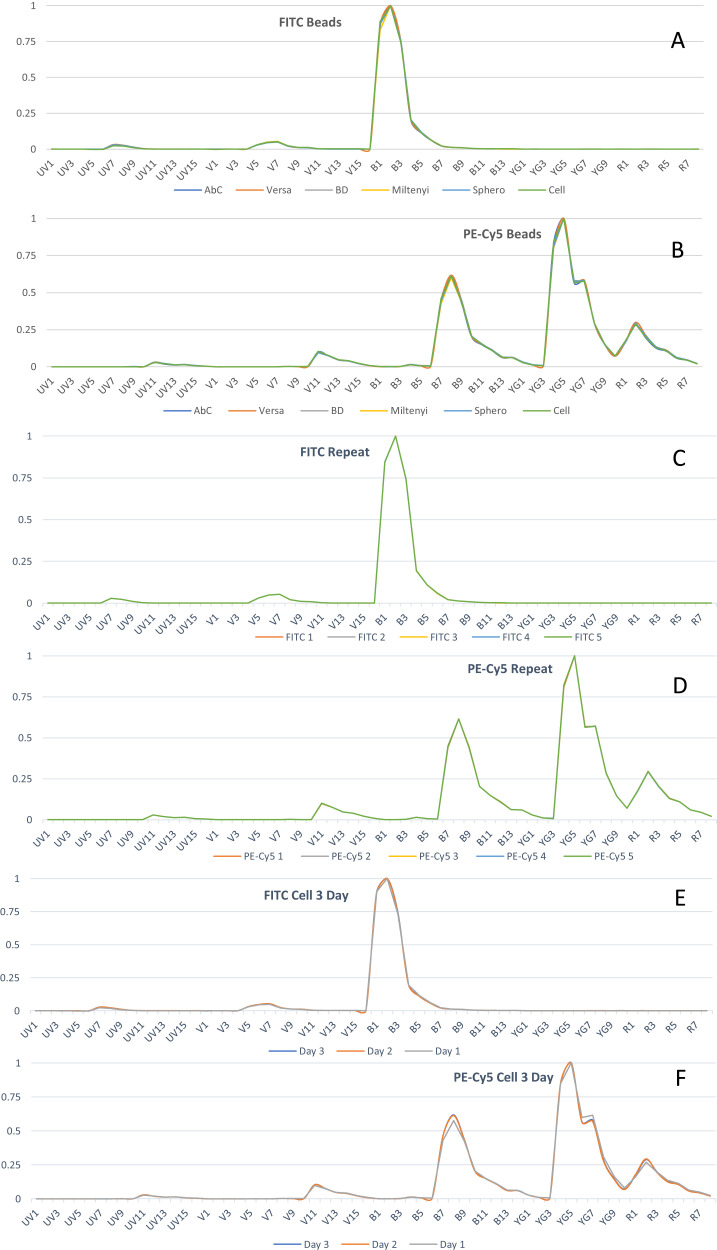
Fluorochrome spectra display slight variations between compensation beads and between days. (**A** and **B**) FITC and PE-Cy5 spectra from cells and beads acquired for Figs. 2 and 4 were overlaid from one run. (**C** and **D**) Multiple FITC and PE-Cy5 spectra were overlaid separately. Spectra from five MACS bead runs from the same day were overlaid. (**E** and **F**) FITC and PE-Cy5 spectra of cells from three different repeats were overlaid.

First, comparisons between beads with the sample fluorochromes were assessed. As shown in Fig. 5A and 5B, emission profiles of FITC and PE-Cy5 are slightly different when comparing different beads. Further testing to determine whether these differences can be identified by the SpectroFlo software revealed that all FITC or PE-Cy5 spectra have similarity indices of 1; thus, for the software, they are identical. Next, assays were performed to determine whether these variations were intrinsic in nature or due to the beads themselves. FITC- and PE-Cy5–stained MACS beads were run five times on the same day and the plots were overlapped, demonstrating perfect overlap (Fig. 5C, 5D).

**FIGURE 6.**
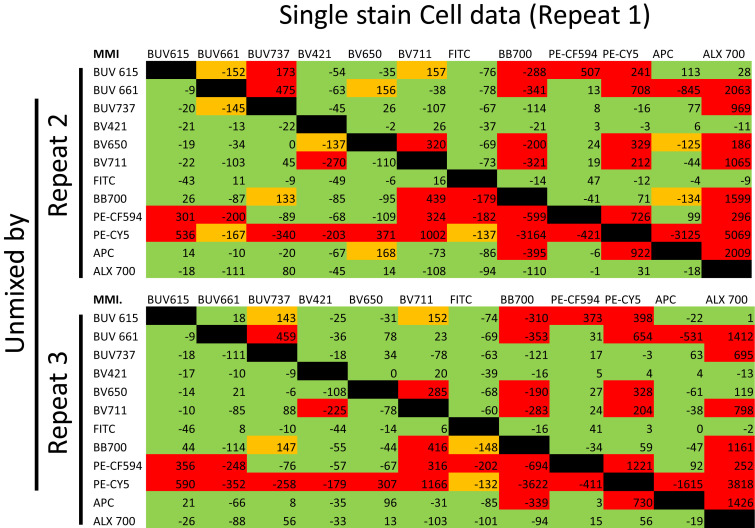
Correction controls and samples must be collected on the same day. PBMC aliquots from the same donor were stained on 3 different days and acquired by flow cytometry. Data from repeats 2 and 3 were used to unmix cell data of repeat 1. Values between −125 and 125 are indicated in green (acceptable), values between −170 and −125 and 125 and 170 are in orange (partly acceptable), and values beyond −170 and 170 are in red (not acceptable). All data are from the Cytek Aurora.

Next, the same fluorochromes staining the same donor cells but on different dates were assessed. Fig. 5E and 5F show that emission profiles (cells only) of FITC and PE-Cy5 are slightly different when comparing three different dates. Next, the quality of unmixing was further assessed. The first set of cell data (repeat 1) was unmixed by the cell data from the other two repeats (with the bright and brighter rule also verified). These data clearly reveal substantial unmixing errors, evident by the presence of significant unacceptable (red) MMI values (Fig. 6). When combined, these data reveal that there are intrinsic differences between independent runs.

### Baseline variation for beads

**FIGURE 7.**
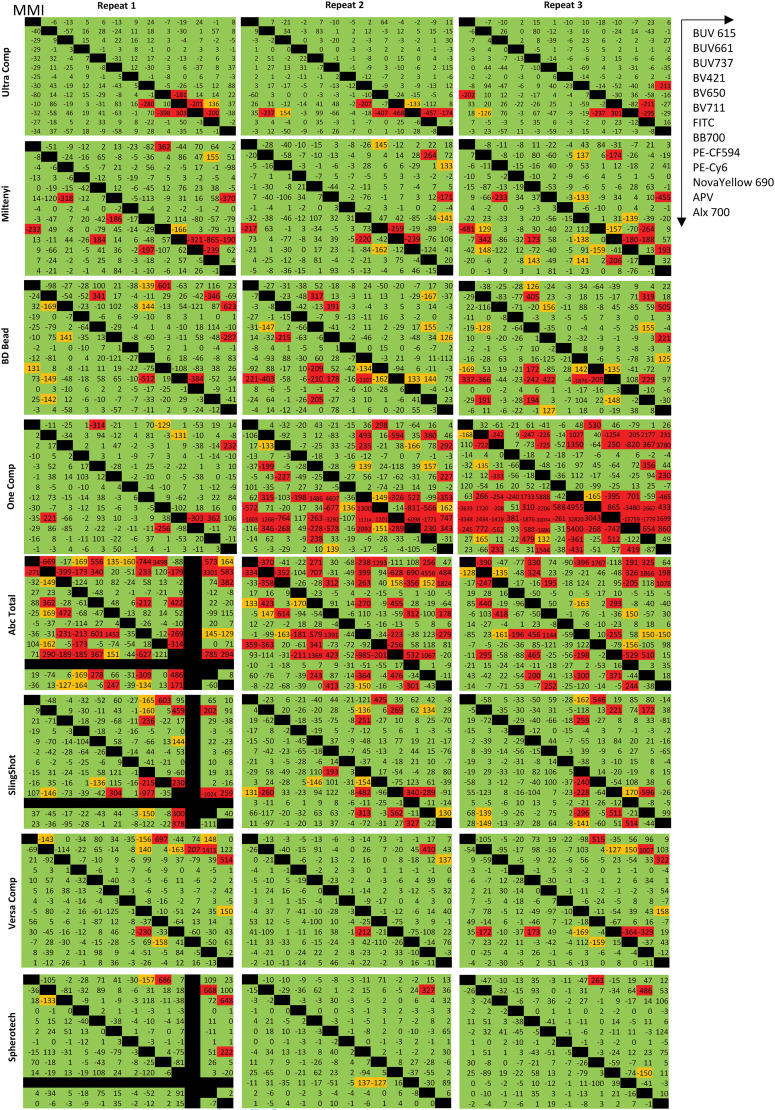
Beads fail to correct themselves. Bead data from Fig. 4 were used to correct the same bead data, with each bead type correcting itself. Values between −125 and 125 are indicated in green (acceptable), values between −170 and −125 and 125 and 170 are in orange (partly acceptable), and values beyond −170 and 170 are in red (not acceptable). All data were acquired on the Cytek Aurora and the BD FACSAria Fusion (*n* = 3).

Next, the same bead single stains were unmixed using the exact same bead single stain samples, similarly to how cell-to-cell unmixing data were assessed above, to assess the amount of baseline variation present in the bead data. These assays generated matrices with considerable numbers of unacceptable (red) categories (Fig. 7). OneComp and AbC Total have a substantial median mismatch, whereas MACS, UltraComp, and BD Biosciences beads have some problems. VersaComp and COMPtrol, which failed bright and brighter for cells, have fewer median mismatch problems in these analyses (this could be due to low MFI because MMI is not a brightness-independent parameter). Finally, Slingshot beads have similar mismatch patterns to MACS, UltraComp, and BD Biosciences beads but failed bright and brighter for some fluorochromes in Fig. 4. These issues were completely unexpected, as these observations have not been previously reported in the literature. To delineate user error or instrument issues within our data, publicly available bead data (15–18) were assessed similarly. The data correction was calculated using the single stain bead data and applied to the same bead data. As the data were generated by different laboratories using different BD FACSymphony A5 cytometers, individual user error and instrument issues were minimized. BD Biosciences beads were used in three instances, whereas the fourth dataset used UltraComp beads. The data (Supplemental Fig. 2) show that these observations are global phenomena.

All of the data so far in this study have been based on assessing single colors with a single abundantly expressed surface protein (CD4) on cells. Next, the differences in correcting using single-color cells or a selection of beads on a small panel of markers were assessed. Abs were used to identify B cells, T cells, monocytes, and CD45RA^+^ cells. These differences are especially evident based on individual marker assessments, where population overcorrelation or undercorrection is evident (Supplemental Fig. 1). Comparisons of cell-based tSNE assessments to one of four bead-based analyses indicate substantial differences in the population distribution variances between correcting using cells or beads (Fig. 3). These data suggest that although unmixing/compensating flow cytometry data using cells is ideal, specific commercially available beads require additional considerations before use.

## Discussion

To investigate how well compensation beads appropriate experimental cells, datasets with cells and eight commercially available Ab-capture compensation beads (beads from BioLegend and Cytek were not released at the time of this study) were generated using 13 commonly used fluorochromes. The literature has not reported any information indicating that these fluorochromes should not be used with beads for proper correction. In this study, we report direct side-by-side comparisons between commercially available beads and primary cells. The bead-based compensation and unmixing data reveal that not all fluorochromes are adequately corrected using beads, which could skew analyses inappropriately.

### Choice of the fluorochrome

The fluorochromes used were specifically chosen to get a high amount of spillover, allowing these assessments. BUV615 and PE-CF594 were expected to have some spillover. BUV661, BV650, PE-Cy5, and allophycocyanin and BUV737, BV711, BB700, Nova Yellow 690, and Alexa Fluor 700 have similar emission maxima per group; hence, cross-laser bleedthrough is expected. FITC and BV421 were negative controls for spillover because they delivered the lowest amount of spillover into other detectors and received the lowest amount from other fluorochromes. Accordingly, FITC and BV421 were expected to always be in an acceptable (green) category, consistent with the data collected (Fig. 4). Fig. 4 shows that some fluorochromes work properly for some detectors, but this depends on the bead brand. For example, the BUV615 detector works well for UltraComp beads, but the rest of the beads assessed had at least one fluorochrome that was not properly corrected for this detector. The PE-CF594 detector generally works well for all beads. The FITC detector worked every time for all bead brands, and the BV421 detector behaves almost similarly. This was unsurprising, as BV421 and FITC were chosen due to their limited spillover into other detectors.

In the choices of primary markers with those fluorochromes, we used only a few Abs, which are well-established primary markers (except CD45RA), but we still found different results between cell- and bead-based unmixing. An interesting result that was unexpected is that of the eight bead sets that were tested, two of them (COMPtrol and VersaComp) failed to elicit brighter signals than those signals on cells, leading to exclusion from this study. Two other bead sets (Slingshot and AbC Total) could only be used to correct five fluorochromes. In a high-parameter panel where researchers are trying to study the role of a new marker, a less defined marker, or a marker in a disease state or in an exploratory type of work, this variability could lead to many issues with interpretation of the data. It is highly likely that in these scenarios, researchers could be misled by the compensation bead-based data correction. This variability leading to differing results could also severely impact the repeatability and rigor of these studies.

In this study, the rules of compensation were followed as best as possible. To preserve the emission profiles of the fluorochromes, Abs were always kept and maintained in the dark and cold, and data were acquired as soon as possible. All beads and cells were stained with the exact same Ab vial to ensure that each assay used the sample fluorochrome without batch differences. Sufficient data were collected to get a statistically robust calculation. Another factor to consider is AF from the beads. AF within cell populations is known to elicit differing AF patterns based on cell types. BD Biosciences, AbC Total, MACS, COMPtrol, and VersaComp provided positive and blank beads in separate vials. To ensure that the AF of the universal negative control and the positive beads remain the same, only the positive bead was used for these cases, making the AF assessment more straightforward. OneComp, UltraComp, and Slingshot provide both beads in the same vials. The users need to ensure that both of these beads have the exact the same AF, the lack of which may introduce an additional source of error in the single stain control data, potentially changing the unmixing or compensation. UltraComp (lot no. 2306368) had this problem, so a new lot of beads were acquired for further experiments.

Users are recommended to check the AF for these beads beforehand. The data herein indicate that users should assess specific bead AF before staining single stain controls and performing flow cytometry experiments to ensure that their marker expressions are not miscalculated by AF mismatch.

Compensation control needs to be brighter than samples. All of the beads used in this study were tested beforehand to determine the Ab amount needed to be saturated for all of the different beads. This ensures that the assays will reach the maximum intensity of the beads. For each dataset, MFI was checked to confirm that the beads were brighter than the cell to fulfill the first rule of compensation/unmixing. This was especially important for COMPtrol and VersaComp beads, as data demonstrated that they generally produce dimmer signals than do cells for CD4. Because of this, they were precluded from further assessments. Of interest, AbC Total and Slingshot beads were brighter for some fluorochromes, but not others. As the Abs used to compare the beads to the cells were against a highly abundant protein (CD4), lower abundant proteins may allow these beads to produce brighter signals than those from the cells. Taken together, these data signify that bead choice is highly dependent on several factors, that is, the AF of the beads, the relative abundance of specific cell markers, and the binding sites on the beads.

What is an acceptable median mismatch? MMI is our choice for quantifying median mismatch. This parameter only measures the median mismatch between the blank and positive populations on the desired secondary detector in terms of the RSD of the blank.

Flow cytometrists have guesstimated the compensation accuracy for years using *N* × *N* plots, a well-accepted technique (4, 6). There is no well-accepted parameter to quantify the degree of median mismatch. This same approach was used in the current study but with the MMI to quantify the median mismatch.

Although the general classifications used in this study are partly subjective, the data in Figs. 4, 6, and 7 and Supplemental Fig. 2 demonstrate well the visualization of the correction problems between cells and beads in combination with fluorochrome usage. Indeed, this is especially seen in assessing CD3 versus CD45RA (Supplemental Fig. 1), where these fluorochromes are inadequately unmixed to different degrees by the beads assessed.

Additional experiments focused on the red categories, which are most easily identifiable and appear double-positive instead of single-positive. This is especially apparent in Fig. 2, where the top right panel with an MMI of 1159 appears as a double-positive population when only one fluorochrome was used in that sample, clearly demonstrating the dangers in improper unmixing/compensation. Correcting the experimental cell data by the same cell data provides the baseline variations for this study because it is using the cells being experimentally analyzed for compensation. The median mismatch is rarely “0,” which was elegantly explained previously by Roederer (22). In all three repeats, the matrices were almost completely green (Fig. 4, row 1). Of all three repeats, out of 468 possible combinations, only one was red, indicating a good correction, which is expected from flow cytometry controls. Similar observations were found from BD Fusion. Interestingly, cell data could not be properly corrected using cell data acquired on different days (Fig. 6), indicating variability in fluorochrome spectra from day to day. This also implies that 1) algorithms are working for both softwares accurately, 2) the gating strategy for correction was working well, 3) cell data can be successfully corrected by the same cells acquired on the same day, and 4) it is possible to correct data generated from these fluorochromes successfully. These results work as positive controls.

Previous studies have used bead-based compensation for cells, but the assumption has always been that the beads are adequately compensation cells. It is abundantly clear from Fig. 4 (rows 2–7) that bead-based correction produces suboptimal results when applied to cells, compared with cell-based controls applied to cells. This means that even with the best effort from researchers, the same experiments/conclusions will be difficult to repeat, which will only fuel the present reproducibility crisis (23–25). At the same time, improper correction can lead to identifying incorrect or biologically nonexistent populations (4).

The issues with bead-based compensation controls on multicolor stains are abundantly clear when comparing our data in Fig. 3 and Supplemental Fig. 1. In this study, we tested four different bead-based unmixing matrices using the bead sets that passed the bright and brighter rule for compensation on one FCS file generated using a multicolor panel. All of the tSNE plots were different. There were several islands that were either missing or newly appeared in the bead-based tSNEs compared with the cell-based tSNE. Indeed, Fig. 3 and Supplemental Fig. 1 clearly show differences in the panels between cells and bead-based compensation. Analyses also represent improper correction leading to population loss and/or gain compared with cell-based correction. The incorrect populations can be easily identified because these markers have been extensively studied and are well characterized. This means that if five different scientists were running the exact same experiment using the exact same protocol, but each used a different bead set for single stain preparation, all five of them would end up with five different results. With improper correction, it is still possible to draw the correct regions/gates if fluorescence minus one controls are used for all fluorochromes (3), but that is not practical to implement. Of note, not every fluorochrome is suboptimal with every compensation bead set, suggesting that a combination of cells and one or more bead set could unmix/compensation data properly.

In general practice, when controls versus experimental samples are compared using tSNE, data are digitally combined and analyzed using these concatenated files before separating the tSNE plots for comparison. This approach was used to compare cell-based versus bead-based correction and revealed many differences between the two. These differences are only present due to the source of correction, suggesting that they are not real and are only artifacts. These findings indicate multiple parameters for which researchers need to account, including bead type and fluorochromes used when assessing gate placement.

Fig. 4 shows that some fluorochromes work properly for some detectors, but this depends on the bead brand. For example, the BUV615 detector works well for UltraComp beads, but the rest of the beads assessed had at least one fluorochrome that was not properly corrected for by this detector. The PE-CF594 detector generally works well for all beads. The BV421 detector works properly for all beads except AbC Total, and the FITC detector worked every time for all bead brands. This was unsurprising, as BV421 and FITC were chosen due to their limited spillover into other detectors.

As mentioned above, single-stained cells could not be correctly corrected using single-stained cells acquired on different days. This implies changes in emission profiles, at least with cells. Next, whether the emission spectra of the same fluorochrome appear differently depending on the bead was assessed. FITC (a primary fluorochrome) and PE-Cy5 (a well-known tandem) were used to assess these potential differences. Both fluorochromes showed slight visual differences in emission profile based on the beads (Fig. 5A, 5B). Based on the rules of compensation, these differences cause the median mismatch in cell data. Similarity index calculation showed that these variations cannot be identified by the SpectroFlo software. Based on the data in Fig. 5C and 5D, it is highly likely that the slight changes in spectra are due to the beads. The best assumption that can be made is that the material used in the bead preparation is somehow interfering with the fluorochrome emission. The mechanism(s) with the bead sets that lead to these disparities is a complex issue. A major impetus to further chemical analyses of the bead sets is associated with potential legal issues with proprietary bead compositions, although better understanding of the interactions of some fluorochromes and some bead sets would improve panel design for researchers.

Recently, Thermo Fisher Scientific warned their users that their UltraComp eBeads are incompatible with BV786 and SB780; they also recommended using cells over UltraComp eBeads for these fluorochromes (26). These data, both from this study and from Thermo Fisher Scientific, reflect that these issues are not unknown to the bead manufacturers. Recently another study compared two beads and found that one worked better than the other (12). Brummelman et al. (27) showed in their figure 1 that single stain cells and single stain beads behave differently upon compensation. Taken together, these data indicate that while unmixing/compensating flow cytometry data using cells are ideal, specific commercially available beads require additional considerations before use.

### Beads failed to unmix themselves

Unlike Fig. 4 (row 1), where cells adequately corrected themselves, beads failed to do this, the extent to which is dependent on the bead type (Fig. 7). COMPtrol beads performed the best with just a few red categories (this could be due to their low brightness). In contrast, AbC Total and OneComp beads performed the worst, with the maximum number of red categories. Based on the current knowledge and dogma, this phenomenon should not exist and (to our knowledge) has not been previously reported. There is currently no explanation in the literature for why it exists. Naturally, this raises the question of how or to what extent data correction using beads can be trusted when they cannot correct themselves. This phenomenon can be recapitulated using publicly available datasets run on different machines (Supplemental Fig. 2). This is not an isolated issue due to technical errors, artifacts from sample preparation or the cytometer, or user error.

Lastly, the approximate number of Ab binding sites on beads compared with each Ab binding site on the cell was calculated (Table II). The numbers were expected to be similar among one type of bead, as the same clone and saturating amount of Ab were used for each stain. Interestingly, binding sites for NovaFluor dyes could not be calculated. For an unknown reason, the binding of this Ab to cells was extremely poor but worked normally with beads, and this pushed the numbers abnormally high; accordingly Nova data were not used for calculations in Table II. The experimental variation was estimated to be roughly within ±10% (acceptable, green), with 10–20% variation (yellow) being partly acceptable. For all bead types, there are some exceptions where the number of Ab binding sites stays outside this range, mainly for fluorochromes such as BV650, FITC, and Alexa Fluor 700. Notably, FITC showed a discrepancy, but this molecule has been used in flow cytometry since the inception of the technology. Regarding beads, VersaComp, COMPtrol, BD Biosciences, and MACS performed best with the lowest number of discrepancies. The performance of these beads indicates that the differences in fluorochromes mostly do not alter the binding ability of Abs. UltraComp, OneComp, and Slingshot have several numbers outside the ±10% cutoff, and AbC Total beads displayed the highest number of discrepancies. These observations suggest that the discrepancies are likely due to the bead shape/geometry or the material itself, which inhibits the Ab binding to the bead. In general, Alexa Fluor 700 has more binding sites for any bead type than any other fluorochrome. Perhaps the real Ab binding to the beads is influencing the fluorochrome to bind to the bead.

Additionally, the beads did not perform the same between machines. In the case of the analyzer, the samples allowed decent, steady events per second for all beads, but the sorter events per second varied. A flow rate of 5 was required for OneComp, UltraComp, and Slingshot beads to get an events per second close to 100; however, a flow rate of 2 was adequate for the other beads. Most likely, the materials used for the beads requiring a higher flow rate are heavy, whereas the rest are lighter. This is likely not happening in the analyzer because the sample line is small, and the beads travel shorter distances compared with the sorter, which has significantly longer sample tubing, requiring a stronger push to eliminate stalls over the longer sample line.

Finally, this study indicates three things that are important for flow cytometry users to know. First, our observations show that creating single-color stains using cells for correction is the best choice. Second, in many cases, where the use of cells is not an option, researchers need to mix and match cells and commercially available beads, until a better material option is developed. Third, we do have one recommendation for shared resource laboratory managers to mitigate this issue to some extent. Core facility managers should consider running single stains of all possible fluorochromes (CD4 kit or similar sources) using different beads, or the beads of choice for the users of the facilities. This database needs all samples to run on the same day. If scatter plots provide sufficient separation, multiple beads can be added in the same tube to reduce the sample number. Different beads can be identified digitally during analyses. At the same time, single color stains for cell types identified by the user base should be analyzed. Once a multicolor panel is finalized, the MMI matrix of cells can be generated. Using a script, s shared resource laboratory can identify which fluorochrome is suitable on which bead to get a MMI matrix as close as cell-based matrices. The script will use all possible combinations of fluorochromes and beads from the database to identify the best MMI matrix with the lowest number of red entries.

## Supplementary Material

Supplemental Figures 1 (PDF)
