# Supplementary material for: Side-by-Side Comparison of Compensation Beads Used in Polychromatic Flow Cytometry
Source: Immunohorizons. 2023 Dec 6;7(12):819–33. doi: 10.4049/immunohorizons.2300066 (PMC10759156; doi:10.4049/immunohorizons.2300066)
Supplement: Supplemental Figures 1 (PDF) [file IH_2300066_Supplemental_1.pdf]

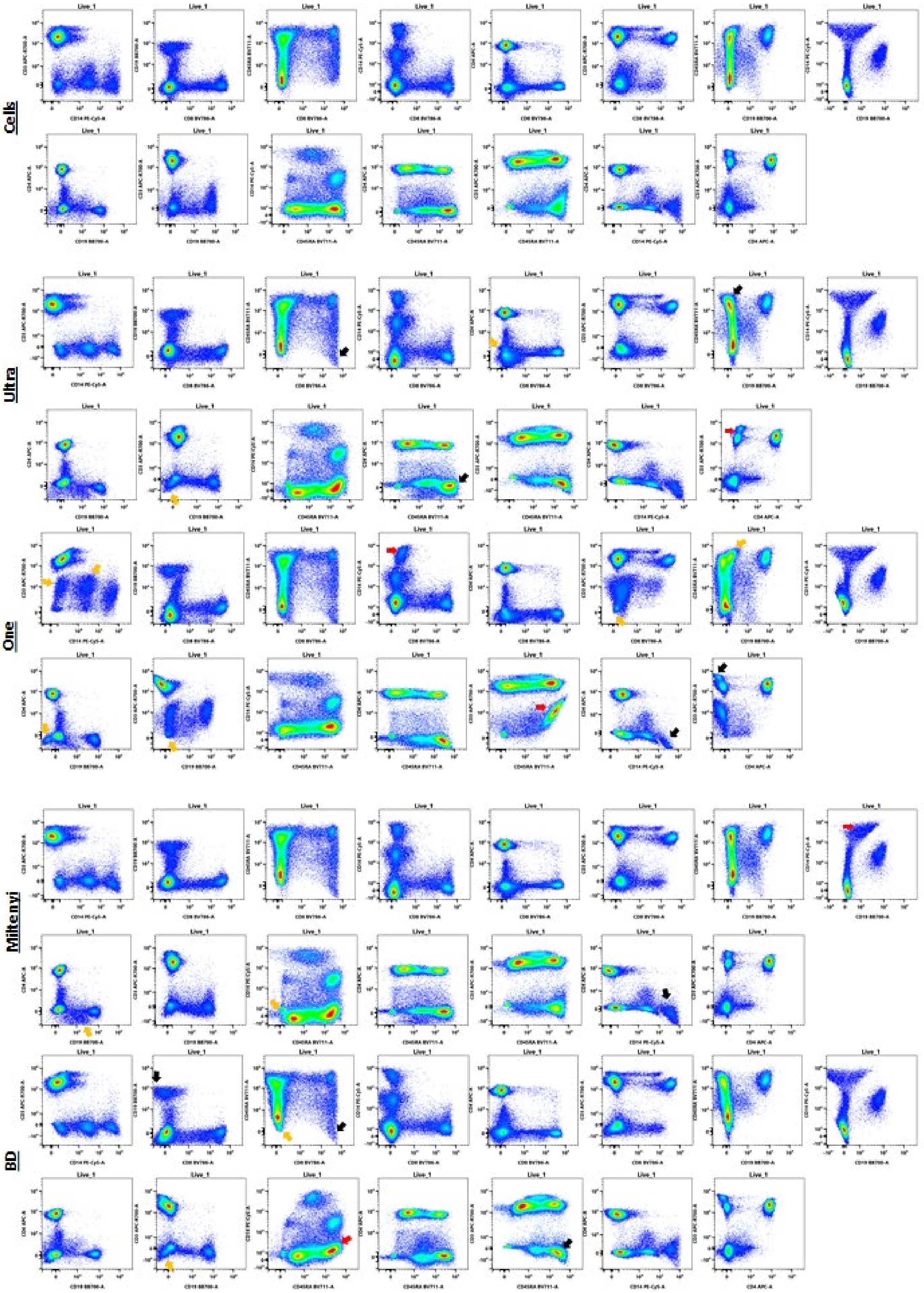

Supplementary Figure 1: Compensation beads alter multicolor stained PBMC profiles.
PBMCs were stained for DAPI, CD3, CD19, CD4, CD8, and CD45RA and assayed by flow cytometry. Single color controls using cells and four different compensation beads were stained and assayed. A representative panel analysis from one data set is shown. Every row represents the same FCS file from the fully stained cell. Single stained cells and beads were used to create and apply the unmixing matrices on the same FCS file. All possible fluorochrome combinations are shown here except DAPI. Red and Black arrows were used to show under and over-corrected populations, while orange arrows represent new/extra populations generated by bead-based correction. All data were acquired on the Cytek Aurora, n=3.

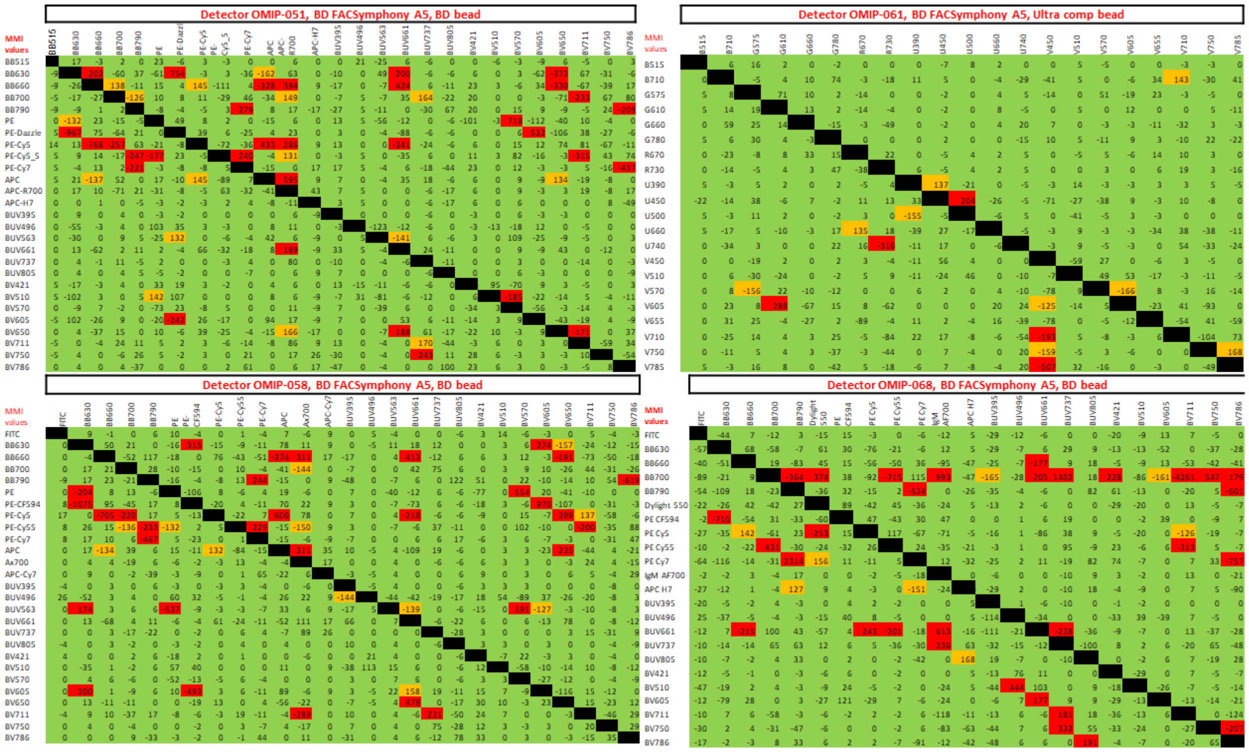

Supplementary Figure 2: Control bead data from published cytometry studies also show inadequate data correction. MMI calculations were performed on single stain bead data found in four different OMIPs. Values between -125 to 125 are indicated by green color (acceptable), values between -170 to -125 and 125 to 170 are in orange (partly acceptable), and values beyond -170 and 170 are in red (not acceptable).
